# Lipopolysaccharide Preconditioning Protects Hepatocytes from Ischemia/Reperfusion Injury (IRI) through Inhibiting ATF4-CHOP Pathway in Mice

**DOI:** 10.1371/journal.pone.0065568

**Published:** 2013-06-04

**Authors:** Jianhua Rao, Jianjie Qin, Xiaofeng Qian, Ling Lu, Ping Wang, Zhengshan Wu, Yuan Zhai, Feng Zhang, Guoqiang Li, Xuehao Wang

**Affiliations:** Liver Transplantation Center, The First Affiliated Hospital of Nanjing Medical University; Key Laboratory of Living Donor Liver Transplantation of Ministry of Public Health, Nanjing, PR China; University of Leicester, United Kingdom

## Abstract

**Background:**

Low-dose lipopolysaccharide (LPS) preconditioning-induced liver protection has been demonstrated during ischemia-reperfusion injury (IRI) in several organs but has not been sufficiently elucidated underlying causal mechanism. This study investigated the role of low-dose LPS preconditioning on ATF4-CHOP pathway as well as the effects of the pathway on tissue injury and inflammation in a mouse model of liver partial-warm IRI.

**Methods:**

LPS (100 µg/kg/d) was injected intraperitoneally two days before ischemia. Hepatic injury was evaluated based on serum alanine aminotransferase levels, histopathology, and caspase-3 activity. The ATF4-CHOP pathway and its related apoptotic molecules were investigated after reperfusion. The role of LPS preconditioning on apoptosis and ATF4-CHOP pathway was examined in vitro. Moreover, the effects of the ATF4-CHOP pathway on apoptosis, Caspase-12, and Caspase-3 were determined with ATF4 small interfering RNA (siRNA). Inflammatory cytokine expression was also checked after reperfusion. Inflammatory cytokines and related signaling pathways were analyzed in vitro in macrophages treated by LPS preconditioning or ATF4 siRNA.

**Results:**

LPS preconditioning significantly attenuated liver injury after IRI. As demonstrated by in vitro experiments, LPS preconditioning significantly reduced the upregulation of the ATF4-CHOP pathway and inhibited Caspase-12 and Caspase-3 activation after IRI. Later experiments showed that ATF4 knockdown significantly suppressed CHOP, cleaved caspase-12 and caspase-3 expression, as well as inhibited hepatocellular apoptosis. In addition, in mice pretreated with LPS, TNF-α and IL-6 were inhibited after reperfusion, whereas IL-10 was upregulated. Similarly, low-dose LPS significantly inhibited TNF-α, IL-6, ATF4-CHOP pathway, NF-κB pathway, and ERK1/2 in high-dose LPS-stimulated macrophages, whereas IL-10 and cytokine signaling (SOCS)-3 suppressor were induced. Importantly, ATF4 siRNA is consistent with results of LPS preconditioning in macrophages.

**Conclusions:**

This work is the first time to provide evidence for LPS preconditioning protects hepatocytes from IRI through inhibiting ATF4-CHOP pathway, which may be critical to reducing related apoptosis molecules and modulating innate inflammation.

## Introduction

Ischemia-reperfusion injury (IRI) is a key contributing factor in liver dysfunction and failure after hepatic trauma, resection, and liver transplantation [1]–[4]. Endoplasmic reticulum (ER) stress is involved in liver damage following ischemia reperfusion (IR) [5]–[6]. The ER is a centrally located intracellular organelle that performs critical functions in protein synthesis and folding, lipid and sterol synthesis, and calcium homeostasis. Perturbations in any of the major functions of the ER result in ER stress, which can be induced by various pathophysiological stimuli, such as hypoxia, glucose deprivation, and calcium depletion from the lumen. IR disturbs cellular energy levels, redox states, and Ca^2+^ concentration, which cause ER stress. ER stress responses are triggered by activation of three distinct signaling pathways mediated by protein kinase R-like ER kinase (PERK), inositol-requiring enzyme (IRE), and activating transcription factor (ATF) 6 [7]–[8]. ER stress is characterized by the accumulation of unfolded and misfolded proteins in the ER lumen that trigger unfolded protein response (UPR), which attempts to relieve ER stress by suppressing protein synthesis, upregulating chaperones, and degrading misfolded proteins [7]–[10]. If unsuccessful, the UPR switches from a cytoprotective response to an apoptotic one in stressed cells [9], [10].

PERK activation triggers eIF2α phosphorylation on serine 51, which increases the translation of the mRNA that encodes ATF4 [11]–[12]. ATF4 has a transcriptional activity over several genes, including C/EBP homologous protein (CHOP), which is a transcription factor that induces apoptosis [12]. ER stress bears UPR, which is an initially adaptive response that can ultimately lead to an execution phase that involves CHOP-mediated apoptosis [13]. Toll-like receptor (TLR) signaling can selectively attenuate translational activation of ATF4 and its downstream target gene, CHOP [14]. Previous studies have shown that TLR functions to maintain tissue homeostasis through regulation of inflammatory response and tissue repair [15]. Of the known TLRs, TLR4 is foremost, and has received particular interest [15], [16]. Lipopolysaccharide (LPS), a principal ligand of TLR4, initiates the TLR4 signaling cascade. A small dose of LPS given systemically confers ischemic protection in the liver, a process that appears to involve inhibition of an inflammatory response [17]. However, the direct effects of a small dose of LPS on hepatocellular apoptosis of liver IRI have not been sufficiently elucidated.

In addition to apoptosis, inflammation is also reportedly initiated by the UPR; the coupling of these responses in specialized cells and tissues is deemed fundamental in the pathogenesis of inflammatory diseases, including liver IRI [6]. Evidence shows that signaling pathways in the UPR and inflammation are interconnected through different mechanisms, including the production of reactive oxygen species and the activation of the transcription factor nuclear factor-κB (NF-κB) and mitogen-activated protein kinase (MAPK) [18]–[21]. TNF-α and IL-6, as proapoptotic cytokines, are two important inflammatory cytokines in the pathogenesis of liver IRI [22]. However, IL-10 is an anti-inflammatoy cytokine and has been demonstrated to reduce liver IRI [23]. NF-κB and MAPK are mainly signaling pathways that regulate these cytokines. The current work examined the effects of ATF4-CHOP pathway on inflammatory cytokine expression (TNF-α, IL-6, and IL-10) and signaling pathways (NF-κB and MAPK).

Results show that first-time inhibition of ATF4-CHOP is critical during low-dose LPS preconditioning against liver IRI: (1) ATF4-CHOP inhibition attenuates hepatocellular apoptosis after reperfusion by suppressing caspase-12 and caspase-3 activation; (2) ATF4-CHOP inhibition reduces inflammatory cytokine expression by repressing pro-inflammatory signaling pathways (NF-κB and MAPK).

## Materials and Methods

### Animals

Male C57BL/6 mice were purchased from the Laboratory Animal Resources of Nanjing Medical University (NJMU). The animals were fed a laboratory diet with water and food and kept under constant environmental conditions, with 12 h light–dark cycles. Procedures were carried out in accordance with the guidelines for the Principles of Laboratory Animal Care and the Guide for the Care and Use of Laboratory Animals. The animal protocol had been approved by the Institutional Animal Care & Use Committee (IACUC) of Nanjing Medical University (Protocol Number NJMU08-092).

### Surgical Procedure and Experimental Design

The present study used segmental (70%) hepatic ischemia model. Briefly, midline laparotomy was performed under 10% chloral hydrate (0.3 g/kg, intraperitoneally) anesthesia in mice. All structures in the portal triad (hepatic artery, portal vein, and bile duct) to the left and median liver lobes were occluded with an atraumatic bulldog clamp for 90 min. The clamp was then removed for reperfusion. The abdomen was immediately closed with a continuous 4-0 silk suture. The mice were sacrificed 6 and 24 h after reperfusion, and blood as well as liver tissue samples were harvested for analysis. Three groups of mice were included to analyze effects of LPS on liver IR. In the sham-operated (sham) group (n = 12), mice were given anesthesia and subjected to laparotomy as well as exposure of the portal triad without hepatic ischemia. In the I/R (IR) group (n = 12), mice were subjected to ischemia and reperfusion as described above. In the LPS preconditioning (LPS PC+IR) group (n = 12), mice were injected intraperitoneally with 100 µg/kg LPS intravenously for two consecutive days before I/R operation. LPS (*Escherichia coli* O111:B4) was purchased from Sigma-Aldrich (Shanghai, China) and diluted in saline to 10 µg/ml. The mice in the sham and I/R groups received an equivalent volume as control. To access effects of ATF4 knockdown on liver IR, ATF4 siRNA and NS siRNA (2 mg/kg) was given intravenously 4 hours prior to ischemia [24]. Reports have previously documented the efficacy of this siRNA approach in the liver, with >40% of intravenously infused siRNA accumulating in the ischemic mouse livers [24], [25].

### Serum Biochemical Examination

Blood samples collected 6 and 24 h after reperfusion was centrifuged to obtain serum. The serum level of alanine aminotransferase (ALT) and lactate dehydrogenase (LDH) was measured to assess the extent of hepatocyte damage using an automated chemical analyzer (Olympus Automated Chemistry Analyzer AU5400, Japan).

### Histopathologic Study

Liver specimens were fixed with 10% neutral formaldehyde and then embedded in paraffin. The specimens were sectioned at 4 µm and stained with hematoxylin and eosin. The sections were used in histopathologic analysis by light microscopy. Sections were scored from 0 to 4 for sinusoidal congestion, vacuolization of hepatocyte cytoplasm, and parenchymal, as described by Suzuki et al. [26].

### Caspase-3 Activity

Caspase-3 activity was checked in liver tissues 6 h after reperfusion. Frozen samples of ischemic tissues were homogenized with a Polytron homogenizer and centrifuged at 16,000 g for 20 minutes. Activity was measured with an assay kit (Calbiochem) according to the manufacturer’s instructions.

### Terminal Deoxynucleotidyl Transferase dUTP Nick End Labeling (TUNEL) Staining

Paraffin sections (4 µm in thickness) were deparaffinized in toluene and then dehydrated through a graded series of ethanol solutions. Sections were stained by TUNEL using a commercially available kit (in situ cell death detection kit, Roche-Boehringer Mannheim, Germany).

### Western Blot Analysis

Proteins were extracted from liver tissues subjected to ischemia or cell lysates, and their concentrations were determined by the Bradford assay (Bio-Rad, CA). About 30 µg of the protein sample was resolved by sodium dodecyl sulfate polyacrylamide gel electrophoresis and transferred to nitrocellulose membranes (Sunshine Biotechnology, China). These membranes were blocked in skim milk powder (5% wt/vol) with phosphate buffered saline with 0.1% Tween 20 (PBS-T) at 4°C overnight. Membranes were then incubated with primary antibodies for CHOP, Caspase-12, Caspase-3, p-NF-κB p65, IκBα, SCOS3, p-ERK1/2, β-actin (Cell Signaling Technology, Danvers, MA), and ATF4 (Santa Cruz Biotechnology, Santa Cruz, CA). Following three washes with PBS-T, the membranes were incubated for 1 h at room temperature with peroxidase-conjugated secondary antibody (Cell Signaling Technology, Danvers, MA). The final results were obtained by exposure to autoradiographic film (Kodak XAR film), and then visualized via a chemiluminescent detection system (ECL Substrate Western blot detection system, Pierce, IL).

### Quantitative Real-time PCR

Reverse transcription reactions were performed using the Super-Script First-Strand Synthesis System (Invitrogen, CA). To determine relative number of cDNA molecules in the reverse transcribed samples, real-time PCR analyses were performed using the Light-Cycler system (Roche, Indianapolis, IN). PCR was performed according to the procedure as previous [19]. PCR was performed using 10 µl 2x Master Mix SYBR Green I (Takara, Japan), 0.25 µM of each 5′ and 3′ primer, and 2 µl samples or H2O to a final volume of 20 µl. Samples were denatured at 94°C for 5 min. Amplification and fluorescence determination were carried out in 3 steps: denaturation at 94°C for 10 sec, annealing at 60°C for 15 sec, extension at 72°C for 20 sec; and at the end of extension, detection of SYBR green fluorescence, which reflects the amount of double-stranded DNA. The amplification cycle number was 45. To discriminate specific from nonspecific cDNA products, a melting curve was obtained at the end of each run. Products were denatured at 95°C for 3 sec, and the temperature was then decreased to 58°C for 15 sec and raised slowly from 58°C to 95°C using a temperature transition rate of 0.1°C/sec. Data were normalized with GAPDH levels in the samples. Primers were designed by Oligo 6.0. Primer sets (sense sequence and antisense sequence, respectively) for the following genes were: HPRT forward, 5′- TCA ACG GGG GAC ATA AAA GT-3′, reverse, 5′- TGC ATT GTT TTA CCA GTG TCA A′; TNF-α forward, 5′- GCC TCT TCT CAT TCC TGC TTG T-3′, reverse, 5′- TTG AGA TCC ATG CCG TTG-3′; IL-6 forward, 5′- GCT ACC AAA CTG GAT ATA ATC AGG A-3′, reverse, 5′- CCA GGT AGC TAT GGT ACT CCA GAA-3′; IL-10 forward, 5′- ACT GCA CCC ACT TCC CAGT -3′, reverse, 5′- TGT CCA GCT GGT CCT TTG TT-3′.

### Immunohistochemistry

Formalin-fixed and paraffin-embedded liver sections with a thickness of 4 µm were dewaxed in xylene and graded alcohols, hydrated, and washed in PBS. After pretreatment in a microwave oven, endogenous peroxidase was inhibited by 3% hydrogen peroxide in methanol for 20 min, followed by avidin-biotin blocking using a biotin-blocking kit (DAKO, Germany). Slides were then incubated with antibody (CHOP) for 4 h in a moist chamber at room temperature, washed in PBS, and incubated with biotinylated goat anti-rabbit/mouse antibody. Slides were developed with the Dako Liquid 3,′3-diaminobenzidine tetrahydrochloride +Substrate Chromogen System and counterstained with hematoxylin. The sections were used in histopathologic analysis by light microscopy.

### Cell Culture and Treatment

Mouse hepatocytes were isolated using a two-step in situ collagenase perfusion procedure [27]. Livers from the C57BL/6 mice were perfused in situ through the portal vein with ethylene glycol tetraacetic acid (EGTA) buffer (0.5 mM EGTA, 137 mM NaCl, 4.7 mM KCl, 1.2 mM KH2PO4, 0.65 mM MgSO4, and 10.07 mM HEPES at pH 7.4) at a flow rate of 5 ml/min for 10 min, followed by collagenase buffer (67 mM NaCl, 6.7 mM KCl, 4.76 mM CaCl2, 0.035% collagenase type II, and 10.07 mM HEPES at pH 7.6) at a flow rate of 5 ml/min for 15 min. After centrifugation, the hepatocytes were collected and seeded in DMEM containing 10% FBS, 100 units/ml penicillin, and 100 µg/ml streptomycin. Cells were preincubated with low-dose LPS (1 ng/ml for 8 h), then TM (10 µg/ml for 24 h), or H_2_O_2_ (200 µm for 24 h) to induce ER and oxidative stress.

Murine BM macrophages were differentiated from bone marrow from 6- to 8-week old C57B/6 mice, by culturing in 1x DMEM, 10% fetal bovine serum, 1% penicillin/streptomycin, and 10% L929-conditioned medium for 6 days. Cell purity was assayed to be 94% to 99% CD11b^+^. Cells were preincubated with low-dose LPS (1 ng/ml for 8 h), then high-dose LPS (1 µg/ml for 3 or 24 h) to analyze signaling pathway or cytokine expression.

### Knockdown of ATF4 Expression using ATF4 Small Interfering RNA (siRNA)-mediated Gene Transfection

Hepatocytes and BM macrophages were grown and transiently transfected with ATF4 siRNA or negative control siRNA using Transfection Reagent Lipofectamine™ RNAiMAX (Invitrogen, CA, USA) according to the manufacturer’s instructions. In brief, cells were seeded at 1×10^6^ per well in 1.5 ml of OPTI-medium (Invitrogen, CA, USA) in a 6-well plate. After 20 h, the cells were transfected with 20 nmol/ml ATF4 siRNA or negative control siRNA. About 6 h after transfection, the medium was changed to a regular medium, and the cells were treated as described above after 24 h.

### Statistical Analysis

Difference among groups was determined for statistical significance using one-way ANOVA or Student’s *t*-test. All *P* values were two-sided, and P<0.05 was considered as statistically significant. Statistical calculations were performed with SPSS (Chicago, IL). Data were presented as mean±standard deviation from at least three independent experiments.

## Results

### Attenuating Liver IRI through Low-dosage LPS Preconditioning

**Figure 1:**
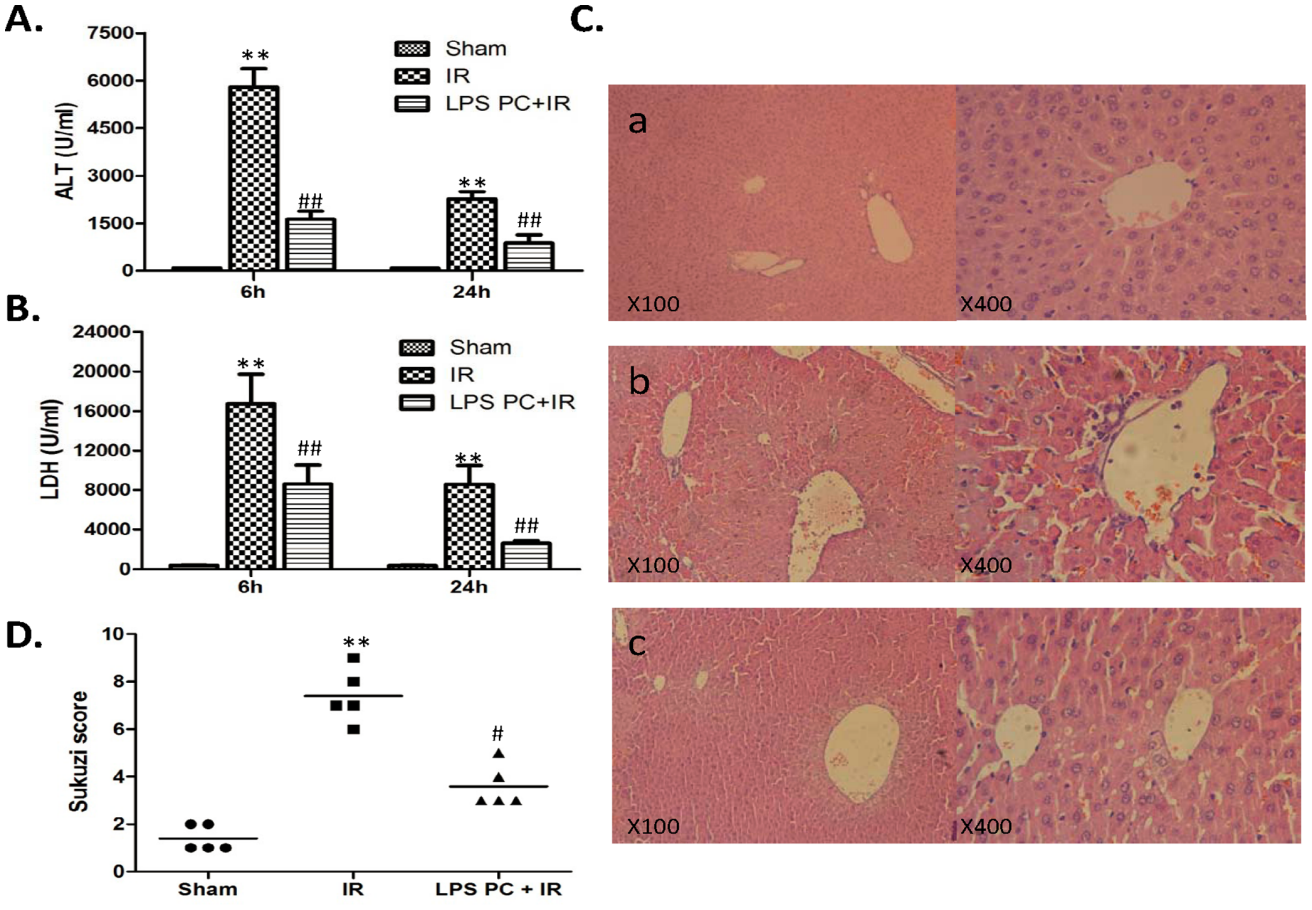
Attenuating liver IRI through low-dosage LPS preconditioning. Mice were subjected to 90 mim of partial liver ischemia, followed by 6 h and 24 h reperfusion. (A) and (B) Hepatocellular function evaluated by ALT (U/L) and LDH (U/L). Mean±SD, **P<0.001 versus sham group; ^##^P<0.001 versus IR group. (C) Histopathalogic analysis of livers harvested 6 hours after reperfusion: (a) Sham group: Normal hepatic architecture; (b) IR group: severe hepatic lobule distortion, sinusoidal congestion, apparent edema, vacuolization and massive necrosis; (c) LPS PC+IR group: mild vacuolization, punctate necrosis and edeman. (D) The severity of liver IRI by Suzuki’s histological grading.

Mice livers were subjected to 90 min of warm ischemia 6 or 24 h after reperfusion. Serum ALT and LDH levels in each group were analyzed (Figs. 1A and B). ALT and LDH levels increased markedly in the I/R group compared with those in the sham group (*P*<0.001). Conversely, when mice were pretreated with LPS intraperitoneally, ALT and LDH levels were significantly decreased compared with those in the I/R control (*P<*0.001). Liver serum enzyme data were consistent with liver pathological analysis (Fig. 1C). The histological parameters observed in the sham, I/R, and LPS preconditioning were according to Suzuki et al [26], and each group was scored as 1.40±0.24, 7.40±0.51, and 3.6±0.40, respectively. These data indicated that LPS preconditioning significantly attenuates IR-induced liver injury.

### Reduction of Hepatocellular Apoptosis after Liver IR through LPS Preconditioning

**Figure 2:**
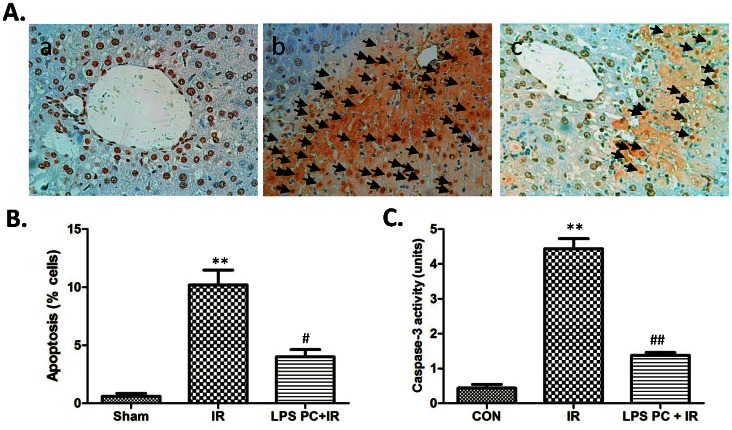
Reduction of hepatocellular apoptosis after liver IR through LPS preconditioning. (A) Liver apoptosis by TUNEL stainning: (a) sham group; (b) IR group and (c) LPS PC+IR group. (B) Apoptotic cells were quantified in six high-power fields (400×), and expressed as percentages of apoptotic cells among total cells. Mean±SD, **P<0.001 versus sham group; ^#^P<0.05 versus IR group. (C) Caspase-3 activity, Mean±SD, **P<0.001 versus sham group; ^##^P<0.001 versus IR group.

Hepatocellular apoptosis was analyzed by TUNEL assay 24 h after reperfusion. TUNEL-positive cells were significantly lower in LPS preconditioned IR liver compared with those in IR control (Fig. 2A). TUNEL-positive cells in the total hepatocytes of three groups were (0.60±0.25)%, (10.20±1.28)%, and (4.00±0.63)%, respectively, indicating that hepatocyte apoptosis was significantly inhibited by LPS preconditioning (Fig. 2B). Apoptotic active caspase-3 directly caused hepatocellular apoptosis after liver IR and reflected the status of apoptosis. Along with the TUNEL assay, Fig. 2C shows that the activity of caspase-3 is significantly inhibited after LPS preconditioning in ischemic liver tissue compared with the IR group (1.38±0.08 and 4.44±0.289, respectively; *P*<0.001).

### ATF4-CHOP Pathway and Related Apoptotic Pathway Inhibition by LPS Preconditioning after IR

**Figure 3:**
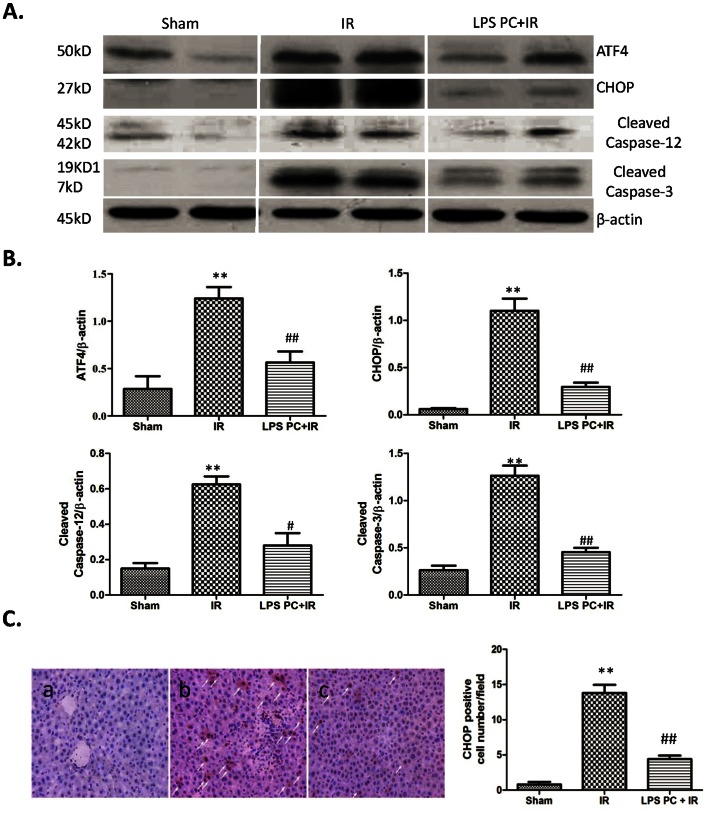
ATF4-CHOP pathway and related apoptotic pathway inhibition by LPS preconditioning after IR. (A) Western-assisted analysis of ATF4, CHOP, Cleaved Caspase-12, Cleaved Caspase-3 and β-Actin. Representative of three experiments. (B) Relative quantities of protein of ATF4, CHOP, Cleaved Caspase-12, Cleaved Caspase-3 to β-Actin, Mean±SD, **P<0.001 versus sham group; ^##^P<0.001 versus IR group; ^#^P<0.05 versus IR group. (C) immunohistochmistry analysis of CHOP: (a) sham group; (b) IR group and (c) LPS PC+IR group. Positive cells were quantified in six high-power fields (400×), and expressed as percentages of positive cells among total cells. Mean±SD,**P<0.001 versus sham group; ^##^P<0.001 versus IR group.

Previous studies have shown that liver IR activates ER stress response, which increases hepatocellular apoptosis [6]. The current work examined the ATF4-CHOP pathway, which is a main apoptotic pathway of ER stress. According to Western blot analysis, liver IR significantly increased ATF4 and CHOP expression, which was obviously inhibited by LPS preconditioning (Figs. 3A and B). As shown in Fig. 3C, the frequency of CHOP positive cells was significantly decreased after LPS preconditioning compared with the IR group (4.40±1.51 and 13.80±1.16, respectively; *P*<0.001), suggesting that LPS preconditioning inhibits the activation of the ATF4-CHOP pathway during liver IRI.

Caspase-12, which is identified as the first ER-associated member of the caspase family, is activated by ER stress. This novel caspase is regarded as a representative molecule involved in cell death-inducing mechanisms relevant to ER stress. Figures 3A and B also show that the expression of cleaved caspase-12 is correlated with the expression of ATF4 and CHOP. Caspase-12 can activate cytoplasmic caspase-3, which affects apoptosis and can directly cause cell death. Thus, cleaved Caspase-3 was analyzed by Western blot, which revealed that caspase-3 activation was significantly lower in the LPS preconditioned group than in the IR group, indicating that ER stress-related apoptosis is also repressed by LPS preconditioning in liver IR.

### ATF4-CHOP Pathway Inhibited by Low-dose LPS Preconditioning in Hepatocytes

**Figure 4:**
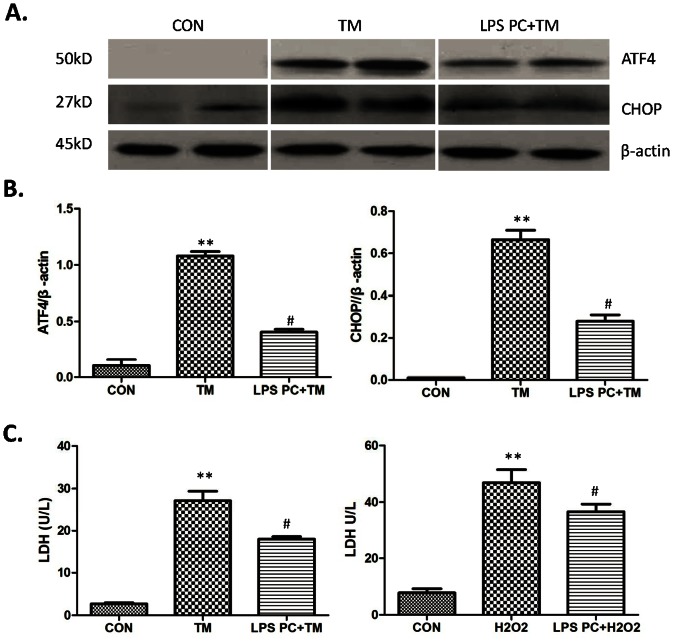
ATF4-CHOP pathway inhibited by low-dose LPS preconditioning in hepatocytes. (A) Western-assisted analysis of ATF4, CHOP and β-Actin. Representative of three experiments. (B) Relative quantities of protein of ATF4 and CHOP to β-Actin, Mean±SD, **P<0.001 versus sham group; ^#^P<0.05 versus IR group. (C)The released LDH level of hepatocytes after TM or H_2_O_2_ treatment, Mean±SD, **P<0.001 versus sham group; ^#^P<0.05 versus IR group.

In addressing the role of low-dose LPS preconditioning on ATF4-CHOP pathway in hepatocytes, primary hepatocytes were previously treated by 10 ng/ml LPS for 8 h, then by 1 µg/ml TM for 6 h. The expression of ATF4 and CHOP was then analyzed by Western blot, which showed that LPS preconditioning significantly repressed ATF4 and TM-induced CHOP (Figs. 4A and B). Furthermore, the direct role of LPS preconditioning on hepatocellular death was studied by the released LDH level of hepatocytes induced by TM or H_2_O_2_ in vitro. Figure 4C shows that LPS preconditioning significantly reduced the released LDH level of hepatocytes after TM (18.01±0.60 and 27.13±2.26, respectively; *P*<0.05) or H_2_O_2_ treatment (36.60±1.37 and 46.93±2.25, respectively; *P*<0.05).

### ATF4 Knockdown Protection against Hepatocellular Death Induced by H_2_O_2_ in vitro

**Figure 5:**
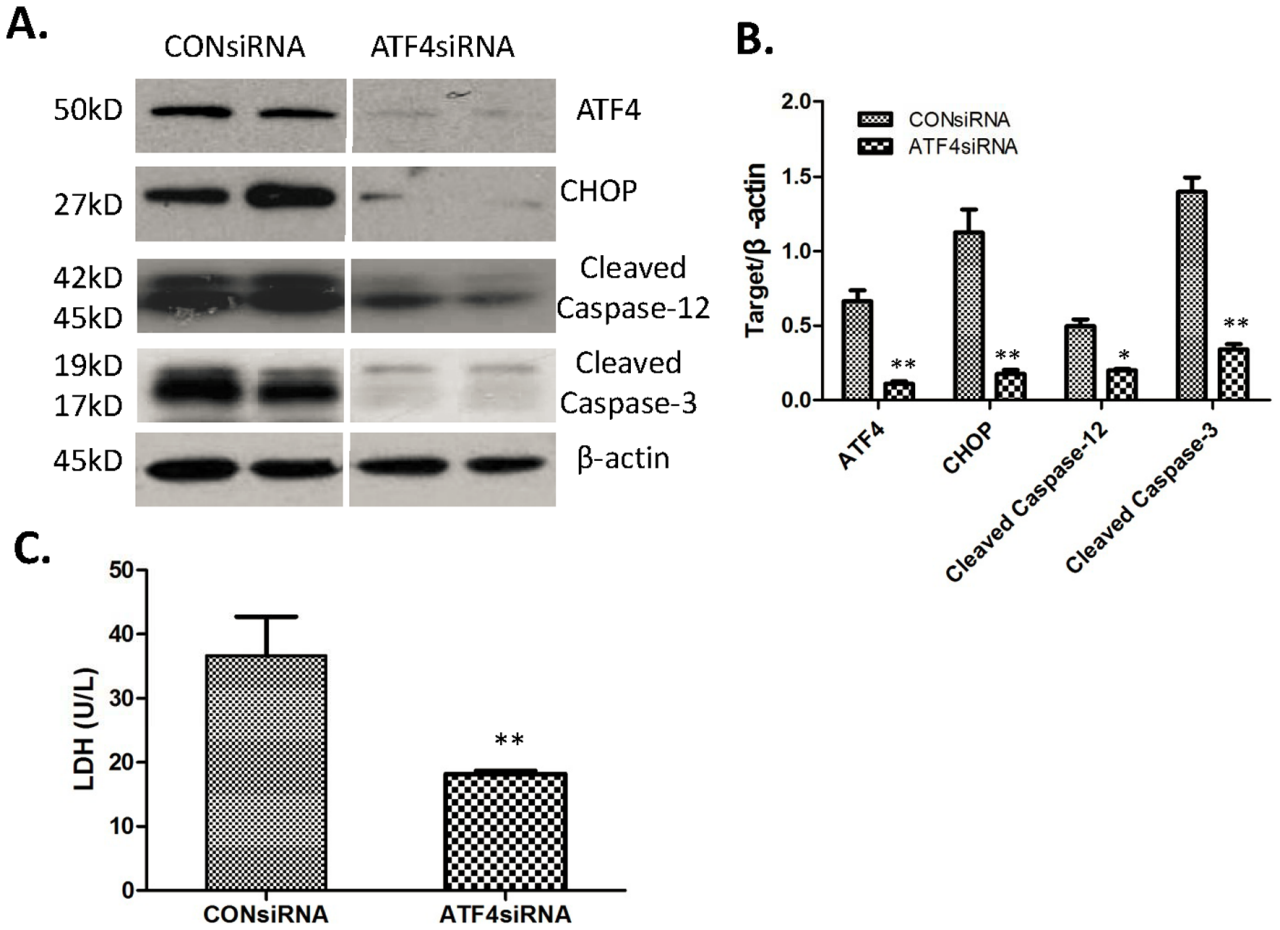
ATF4 knockdown protection against hepatocellular death induced by H_2_O_2_ in vitro. (A) Western-assisted analysis of ATF4, CHOP, Cleaved Caspase-12, Cleaved Caspase-3 and β-Actin. Representative of three experiments. (B) Relative quantities of protein of ATF4, CHOP, Cleaved Caspase-12 and Cleaved Caspase-3 to β-Actin, Mean±SD, **P<0.001 or *P<0.05 versus CONsiRNA. (C)The released LDH level of hepatocytes after H_2_O_2_ treatment, **P<0.001 versus CONsiRNA.

This work explored whether ATF4 expression manipulation using ATF4 siRNA provides protection against hepatocellular death induced by H_2_O_2_. Firstly, primary hepatocytes in ATF4 siRNA were transiently transfected. Second, ATF4 protein expression was significantly suppressed, compared with the cells of the negative control (Figs. 5A and B), indicating that the knockdown of ATF4 expression was successful in liver cells. Then, related protein expressions (CHOP, cleaved caspase-12, and cleaved caspase-3) were further examined, which showed that the knockdown of ATF4 also suppressed CHOP, cleaved caspase-12, and cleaved caspase-3 as induced by H_2_O_2_ treatment (Figs. 5A and B). Next, the released LDH level of hepatocytes was checked in the supernatant after H_2_O_2_ treatment for 24 h (Fig. 5C). Compared with the control siRNA, ATF4 knockdown significantly reduced the LDH level (36.66±6.08 and 18.18±0.52, respectively; *P*<0.05). The repression of ATF4 expression was observed to attenuate the induction of related protein apoptosis.

### ATF4-CHOP Pathway is Involved in Modulating Immune Responses of LPS Preconditioning

**Figure 6:**
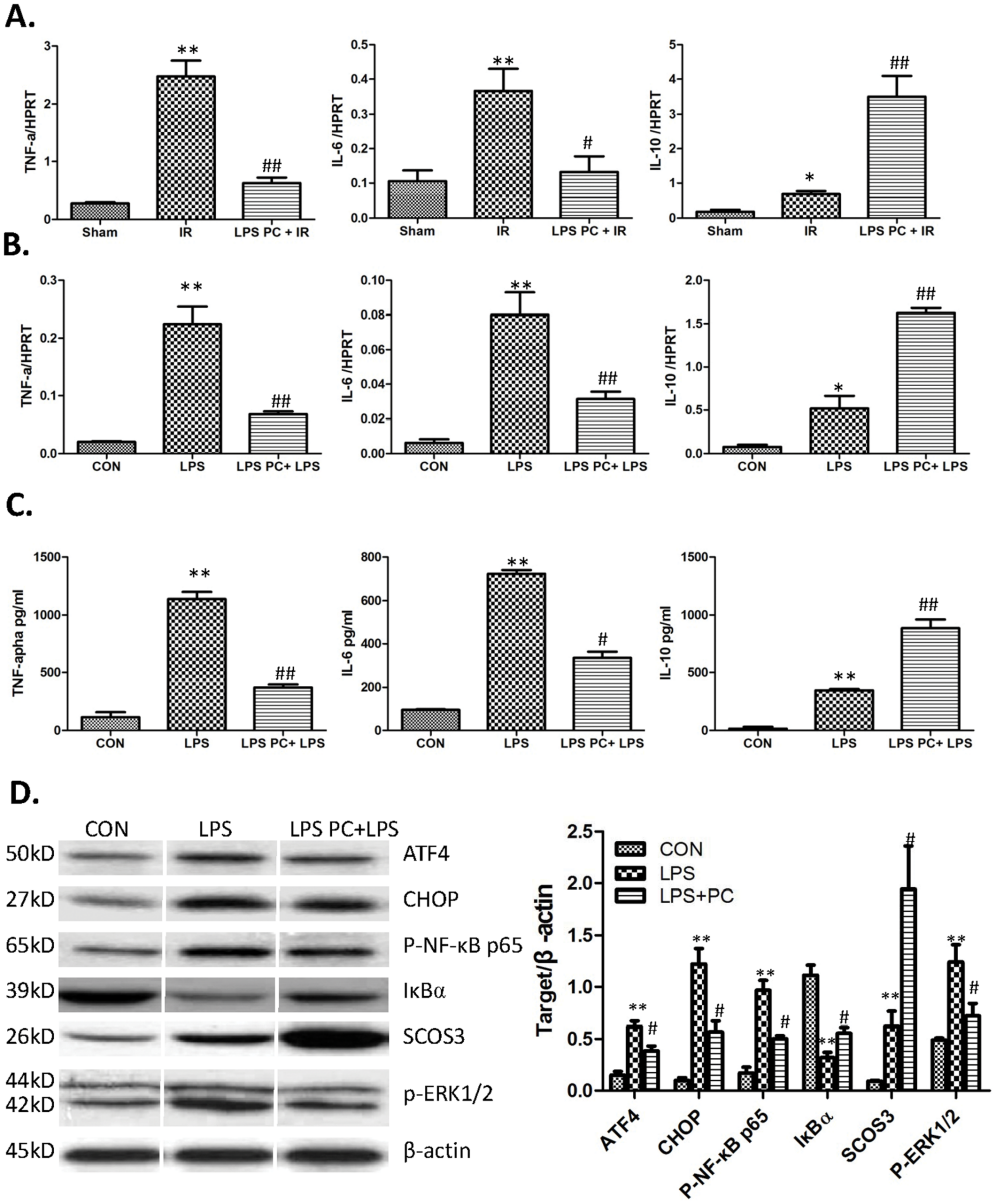
Role of ATF4-CHOP pathway in modulating immune responses of LPS preconditioning. (A) Cytokine gene (TNF-α, IL-6 and IL-10) expression in livers harvested 6 hours after reperfusion by Quantitative RT-PCR analysis. Mean ± SD, **P<0.001 versus sham group; ^##^P<0.001 versus IR group; ^#^P<0.05 versus IR group. (B) Cytokine gene (TNF-α, IL-6 and IL-10)expression in BM-macrophage stimulated by 1 µg/ml LPS treatment for 3 hours. Mean ± SD, **P<0.001 versus CON group; *P<0.05 versus CON group, ^##^P<0.001 versus LPS PC+LPS group. (C) Cytokine secretion (TNF-α, IL-6 and IL-10) by ELISA in BM-macrophage stimulated by 1 µg/ml LPS treatment for 24 hours. Mean ± SD, **P<0.001 versus CON group; *P<0.05 versus CON group, ^##^P<0.001 versus LPS PC+LPS group. (D) Western-assisted analysis of ATF4, CHOP, P-NF-κB p65, IκBα, SCOS3, p-ERK1/2 and β-Actin. Representative of three experiments. The relative quantities of protein to β-Actin, Mean±SD, **P<0.001 versus CON group, ^#^P<0.05 versus LPS PC+LPS group.

A large quantity of inflammatory cytokines is involved in hepatocellular apoptosis and necrosis after IR. TNF-α and IL-6 showed a proapoptotic role during liver IR. On the other hand, IL-10, an anti-inflammatory cytokine, was demonstrated to reduce liver IRI. In further assessing the hepatoprotective effects of LPS preconditioning, mRNA expressions of TNF-α, IL-6, and IL-10 were analyzed in ischemic liver after 6 h of reperfusion by real-time polymerase chain reaction. Figure 6A shows a significantly lower level of TNF-α (0.62±0.10 and 2.47±0.28, respectively; *P*<0.001) and IL-6 (0.13±0.05 and 0.37±0.06, respectively; P<0.05) in the LPS preconditioning group compared with that in the IR group. By contrast, IL-10 expression was significantly higher in the LPS pretreatment group than in the IR group (0.67±0.08 and 3.51±0.58, respectively; *P*<0.001). These data indicated that LPS preconditioning inhibited the expression of proapoptotic cytokines and induced the expression of anti-inflammatoy cytokines during liver IR. BM-macrophages were previously treated by 1 ng/ml LPS for 8 h, then by 1 µg/ml LPS treatment for 3 h for real-time polymerase chain reaction and 24 h for ELISA to assess the modulating role of LPS pretreatment on immune responses. Fig. 6B shows that LPS preconditioning significantly reduced TNF-α and IL-6 expression as well as upregulated IL-10 expression. The secretion of these cytokines in the supernatant was in line with cell mRNA expression (Fig. 6C).

ATF4-CHOP is reported to be involved in many inflammation responses. Herein, the ATF4-CHOP pathway was analyzed during LPS preconditioning on macrophages in vitro. Fig. 6D shows that low-dose LPS preconditioning significantly repressed the ATF4-CHOP pathway induced by high-dose LPS. Activation of the transcription factor NF-κB is involved in the transcriptional regulation of TNF-α and IL-6 in various immune systems [28], [29]. This paper also determined the effects of LPS preconditioning on activation of the NF-κB pathway. As Fig. 6D shows, the expression of p-NF-κB p65 was markedly suppressed, and the degradation of IκBα was partially blocked after LPS preconditioning. In addition, MAPK signaling pathways have been reported to mediate TNF-α and IL-6 in many biological systems. Previous studies have shown that ER-stress can activate ERK1/2 [30]. Herein, the expression of p-ERK1/2 was likewise investigated, which indicated low-dose LPS preconditioning reduced phosphorylation of ERK1/2 induced by the high dose LPS. Previous reports indicated that LPS preconditioning can inhibit NF-κB and MAPK signaling pathways. SOCS3, as a principal negative feedback inhibitor, was studied to elucidate molecular mechanisms. Fig. 6D shows that low-dose LPS preconditioning markedly upregulated SCOS3 expression after high-dose LPS treatment (1.94±0.42 and 0.62±0.14, respectively; *P*<0.05).

### Knockdown of ATF4 Inhibits Inflammatory Response Induced by LPS in vitro

**Figure 7:**
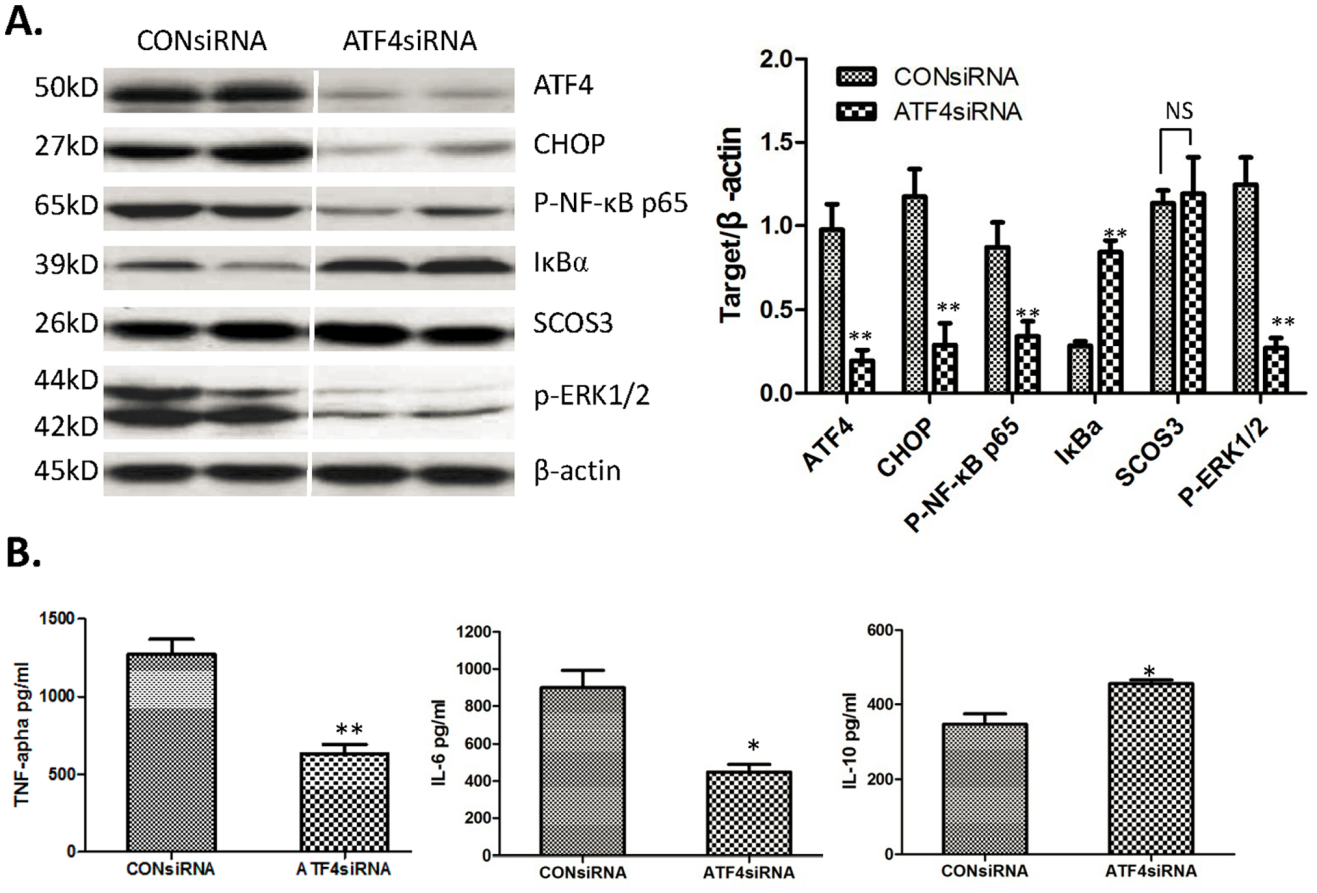
ATF4 knockdown inhibition of immune response in vitro. (A) Western-assisted analysis of ATF4, CHOP, P-NF-κB p65, IκBα, SCOS3, p-ERK1/2 and β-Actin. Representative of three experiments. The relative quantities of protein to β-Actin, Mean±SD, **P<0.001 versus CON group. (B) Cytokine secretion (TNF-α, IL-6 and IL-10) by ELISA in BM-macrophage stimulated by 1 µg/ml LPS treatment for 24 hours. Mean ± SD, **P<0.001 or *P<0.05 versus CONsiRNA group.

A siRNA approach was used to determine whether the transcription factor ATF4 contributes to the inflammatory gene expression in macrophages. The ATF4-CHOP pathway was selectively disrupted. Macrophages lacking ATF4 were then treated with LPS, and analyzed for signaling changes in NF-κB, ERK1/2, and SCOS-3, whose expression of inflammatory genes and secretion of cytokines may be induced by high-dose LPS. Oligo-based siRNA targeting of ATF4 effectively reduced expression of ATF4 protein after LPS treatment (Fig. 7A). ATF4 silencing resulted in a significant decrease in CHOP expression. Next, p-NF-κB p65, IκBα, p-ERK1/2, and SCOS-3 were also checked by Western blot analysis. As shown in Figure 7A, ATF4 knockdown significantly inhibited p-NF-κB p65 and p-ERK1/2 expression, partially blocked IκBα degradation, and slightly increased SCOS-3 expression. In further analyzing the effects of ATF4 knockdown on TNF-α, IL-6, and IL-10, the supernatant of ATF4 knockdown macrophages with 1 ug/ml LPS treatment was examined. ATF4 knockdown markedly reduced the secretion of TNF-α (635.10±54.92 and 1225.01±94.02, respectively; *P*<0.05) and IL-6 (488.20±41.56 and 899.70±92.61, respectively; *P*<0.05), and mildly increased IL-10 (455.6±10.04 and 348.9±25.51, respectively; *P*<0.05) (Fig. 7B). These results indicate that ATF4 knockdown negatively affected TLR4 driven inflammatory response by repression of NF-κB and MAP kinase signaling.

### Knockdown of ATF4 Improves Hepatocellular Function and Ameliorates Liver IRI

**Figure 8:**
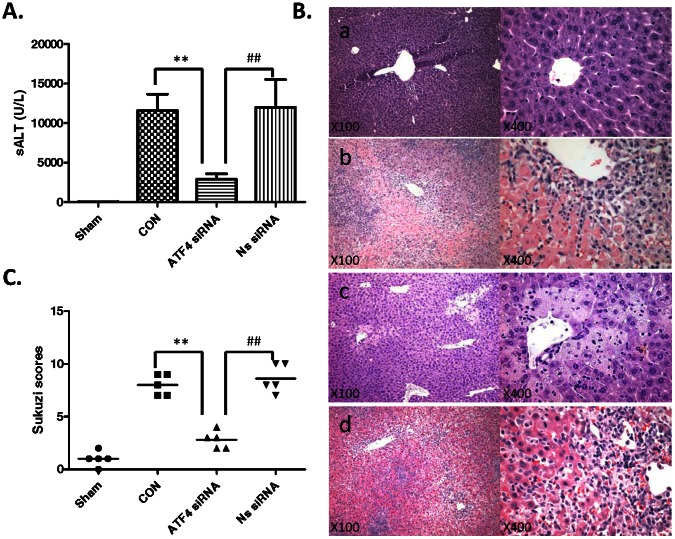
Knockdown of ATF4 improves hepatocellular function and ameliorates liver IRI. Mice were subjected to 90 mim of partial liver ischemia, followed by 6 h reperfusion. (A) Hepatocellular function evaluated by ALT (U/L). Mean±SD, **P<0.001; ^##^P<0.001. (B) Histopathalogic analysis: (a) Sham group: normal hepatic architecture; (b) CON group: severe hepatic lobule distortion, sinusoidal congestion, apparent edema, vacuolization and massive necrosis; (c) ATF4 siRNA group: mild vacuolization, punctate necrosis and edeman; and (d) Ns siRNA: severe hepatic lobule distortion, sinusoidal congestion, apparent edema, vacuolization and massive necrosis. (C) The severity of liver IRI by Suzuki’s histological grading. Mean±SD, **P<0.001; ^##^P<0.001.

We analyzed the hepatocellular function in mouse livers subjected to 90 minutes of warm ischemia followed by 6 hours of reperfusion. As shown in Fig. 8A, sALT levels in mice pretreated with ATF4 siRNA were decreased (*P*<0.001) compared with untreated or NS siRNA-treated controls (2894±340 U/L versus 11580±1039 U/L and 11990±1761 U/L, respectively). These data correlated with Suzuki’s histological grading of liver IRI (Fig. 8B, C). Indeed, knockdown of ATF4 resulted in minimal liver sinusoidal congestion, vacuolization without edema, or necrosis (Fig. 8B(c); score = 2.8±0.37). In contrast, livers in untreated or NS siRNA-treated mice revealed moderate to severe edema and extensive hepatocellular necrosis (Fig. 8C, panels b and d; score = 8.0±0.44 and 8.6±0.60, respectively; P<0.001).

## Discussion

LPS preconditioning, which is recognized to provide resistance to tissue injury caused by a second treatment of LPS or stimulation other than LPS, has been extensively reported in various organs, such as the brain, heart, pancreas, kidney, retina, and liver [31]–[36]. The mechanism and key molecules of LPS preconditioning connected with tissue protection have been reported in the heart, kidney, retina, and liver. LPS is known to produce nitric oxide through the expression of the inducible nitric oxide synthase (iNOS) gene. iNOS plays an important role as a trigger for protective mechanisms associated with LPS preconditioning in the heart and retina [32], [35]. Upregulation of the specific negative regulators SOCS-1, SOCS-3, and IRAK-M of the TLR4 signaling pathway by LPS pretreatment inhibits the inflammatory response after hepatic I/R through suppression of NF-κB and JNK [37]. In addition, IL-10, an anti-inflammatory cytokine, has also been reported to play a central role in renal LPS preconditioning [34]. TLR activation has been found to suppress the ATF4-CHOP pathway of unfolded protein response, which promotes apoptosis and inflammation in many pathological processes [14]. The present study aimed to elucidate whether the ATF4-CHOP pathway is inhibited by a low dose of LPS during liver IRI, and if so, to determine the effects of ATF4-CHOP inhabitation on apoptosis and inflammation.

In this paper, we first studied the protective role of low-dose LPS preconditioning on liver IRI [38], [39]. Mice were injected intravenously with 100 µg/kg LPS intraperitoneally for two consecutive days, and then the liver IRI model was prepared. Liver enzymes (ALT and LDH) were used as biochemical markers of liver injury, through which evidence for the protective effect of LPS preconditioning was found. In line with the biochemical findings, the histological study of the liver indicated that LPS preconditioning improved the liver histological changes induced by I/R. Suzuki scores of hepatic histology were significantly reduced after reperfusion by LPS preconditioning (Fig. 1). Consistent with published data on liver IRI models, LPS preconditioning effectively ameliorated liver injury [36], [37].

Apoptosis and necrosis are central mechanisms of cell death in liver IRI that directly indicate cell death conditions. This work analyzed apoptotic cells in ischemic liver 24 h after reperfusion by TUNEL assay. Fig. 2A shows that the positively stained cells significantly increased in the IR group. The mean number of positively stained cells detected in the LPS preconditioning group was 39.21% that in the IR group (Fig. 2B). Then, caspase-3 activity of liver tissue was examined 6 h after reperfusion, which also reflected the condition of apoptotic cells in ischemic liver. In line with the TUNEL assay, LPS preconditioning significantly inhibited caspase-3 activity elevation after reperfusion (Fig. 2C).

Prolonged and excessive ER stress can cause cell apoptosis. I/R disturbs cellular energy levels, redox states, and Ca_2_^+^ concentration, which cause ER stress. Hepatocytes are characterized by profuse and well-developed ER, which are essential for functions such as protein synthesis and metabolism. Previous studies have shown the involvement of ER stress during IR in various organs, such as heart, kidney, lung, brain, and liver [6], [30]–[43]. ER stress responses are triggered by the activation of three distinct signaling pathways mediated by PERK, IRE-1, and ATF 6. So far, although the ER stress-mediated apoptotic pathway is only partially characterized, some ER stress-specific components of the pathway have been identified. CHOP, also known as growth-arrest-and DNA-damage-inducible gene 153, was originally identified in response to DNA damage [44]. CHOP is reported to be mainly activated by the ATF4 pathway [14], [45], [46]. In fact, CHOP is a proapoptotic transcription factor that is expressed at very low levels under physiological conditions but strongly induced during severe ER stress [45], [46]. However, the ATF4-CHOP pathway is probably involved in apoptosis and necrosis of hepatocytes during IRI. ATF4 and CHOP expression in ischemic liver was checked via Western blot analysis. Figures 3A and B show that ATF4 and CHOP protein expressions were significantly upregulated by IRI. Interestingly, the upregulation of ATF4 and CHOP was significantly inhibited by LPS preconditioning. ER stress-related apoptosis molecules were checked to analyze antiapoptosis of LPS preconditioning during liver IRI. Caspase-12, a murine protein associated with the cytoplasmic face of the ER membrane, has been proposed as an initiator caspase and as an important molecule in the death-driving force in ER stress [47]. Previously, caspase-12 cleavage has been observed in hippocampal neurons that lack the calcium-binding protein hippocalcin [48]. Nakagawa also showed that cells lacking caspase-12 are resistant to degeneration induced by the amyloid β peptide and by TM, which cause ER stress [49]. As a caspase initiator, caspase-12 cleavage can activate caspase-9, caspase-6, and caspase-3. Caspase-3 is an executioner caspase, the activation of which has been reported to induce apoptosis by ER stress [50], [51]. Cleavage caspase-12 and caspase-3 of ischemic liver were then considered. Results showed that liver IR significantly increased the activation of caspase-12 and caspase-3, which was significantly inhibited by LPS preconditioning (Fig. 3A and B). CHOP was further analyzed by inmunohistochemstry, which showed that the number of positive cells was higher in the IR group compared with sham group and was reduced by LPS preconditioning. These results indicated that LPS preconditioning significantly inhibited the activation of ATF4, CHOP, caspase-12, and caspase-3 of ischemic liver.

A hepatocyte-death model in vitro was established by treating primary hepatocytes with TM or 200 umol H_2_O_2_ to assess the effects of LPS preconditioning on apoptosis of hepatocytes directly. After 8 h treatment with 10 ng/ml LPS and 24 h treatment with 10 µg/ml TM or 200 umol H_2_O_2_, the supernatant was examined for LDH to determine cell death conditions. Results showed that LPS preconditioning significantly reduced cell apoptosis induced by TM or H_2_O_2_ (Fig. 4A). In line with previous results, LPS preconditioning was found capable of protecting mesenchymal stem cells (MSCs) from oxidative stress-induced apoptosis and improving the survival of MSCs [52]. Next, this work further analyzed whether LPS preconditioning can inhibit the expression of ATF4 and CHOP induced by TM in hepatocytes in vitro using Western blot. Fig. 4B and C show that LPS preconditioning significantly suppressed ATF4 and CHOP expression compared with the TM group. The direct role of LPS preconditioning in hepatocytes and molecular signaling was determined using this cell death model. In further determining the role of ATF4-CHOP pathway during apoptosis of hepatocytes induced by oxidative stress, ATF4 was subjected to knockdow using ATF4 siRNA. Fig. 5A and B show the successful knockdown of ATF4 compared with CON siRNA; CHOP, cleaved caspase-12, and cleaved caspase-3 were also significantly inhibited following ATF4 siRNA. The results further indicated that ATF4-CHOP pathway directly regulated caspase-12 and caspase-3 activity. Next, transfected hepatocytes with ATF4 siRNA or CON siRNA were treated by 200 µmol H_2_O_2_ for 24 h, which showed that ATF4 knockdown significantly reduced cell death compared with CON siRNA. These data clearly imply that the ATF4-CHOP pathway and subsequent molecules are directly involved during oxidative stress-induced apoptosis. LPS preconditioning offers MSCs protection against oxidative stress-induced apoptosis via the TLR4 and PI3K/Akt pathway [52].

Inflammatory response plays a pathogenic role in liver I/R injury, especially innate immune responses involved in cytokines, including TNF-α, IL-6, IL-10, and so on [2], [6], [22], [37]. Current data demonstrated that LPS preconditioning significantly reduced TNF-α and IL-6 expression in ischemic liver tissue, and increased IL-10 after reperfusion. Consequently, the above results were further demonstrated in vitro. LPS preconditioning inhibited inflammatory cytokines and induced anti-inflammatory cytokines by upregulating specific negative regulators SOCS-1, SOCS-3, and IRAK-M of the TLR-4 signaling pathway [37]. ATF4-CHOP is reportedly involved in many inflammation responses, including innate immune response [14]. Thus, the effects of low-dose LPS preconditioning on ATF4-CHOP pathway induced by high dose LPS was analyzed, and the analysis demonstrated that low-dose LPS preconditioning could partially inhibit the ATF4-CHOP pathway. In distinguishing the regulation roles of the ATF4-CHOP pathway during inflammatory procedure, p-NF-κB 65, IκBα, SCOS3, and p-ERK1/2 were also examined. Subsequently, LPS preconditioning was found to inhibit the expression of ATF4, CHOP, p-NF-κB 65, and p-ERK1/2, as well as increase the expression of IκBα and SCOS3. In further analyzing whether the ATF4-CHOP pathway contributes to inflammatory factors in macrophages, siRNA was used for ATF4 knockdown, which demonstrated that ATF4 silencing reduced the expression of CHOP, p-NF-κB 65, and p-ERK1/2, and increased the expression of IκBα. However, the ATF4-CHOP pathway mildly affected SOCS3 expression. Thus, ATF4 knockdown inhibited inflammatory signaling. In addition, TNF-α, IL-6, and IL-10 were examined in the supernatant to assess the direct effects of ATF4 knockdown on inflammatory cytokines; results were consistent with changes in inflammatory signaling. ATF4 knockdown significantly reduced TNF-α and IL-6 levels, and increased IL-10.

To directly access effects of ATF4-CHOP pathway on liver IR, we disrupted ATF4-CHOP pathway prior to ischemia by using ATF4 siRNA. Our results show that knockdown of ATF4 significantly improves hepatocellular function and ameliorates liver IRI.

In conclusion, this study indicates for the first time that inhibition of the ATF4-CHOP pathway is critical in low-dose LPS preconditioning protection against liver IRI. The inhibition of this pathway not only prevents hepatocellear apoptosis by inhibiting of caspase-12 and caspase-3 activation, but also reduces inflammatory response by suppressing NF-κB and MAPK inflammatory signaling pathways. The present findings provide a novel molecular mechanism of LPS pretreatment protection against hepatocytes injury induced by liver IRI. As such, the ATF4-CHOP pathway may be a novel therapeutic target in liver surgery.
